# Longitudinal sequencing in intramuscular coordination: A new hypothesis of dynamic functions in the human rectus femoris muscle

**DOI:** 10.1371/journal.pone.0183204

**Published:** 2017-08-17

**Authors:** Christoph von Laßberg, Julia A. Schneid, Dominik Graf, Felix Finger, Walter Rapp, Norman Stutzig

**Affiliations:** 1 Department of Sports Medicine, Medical Clinic, University of Tübingen, Tübingen, Germany; 2 Institute of Sports Science, University of Tübingen, Tübingen, Germany; 3 Institute of Sports and Sport Science, University of Freiburg, Freiburg, Germany; 4 Department of Sport and Motion Science, University of Stuttgart, Stuttgart, Germany; Semmelweis Egyetem, HUNGARY

## Abstract

The punctum fixum-punctum mobile model has been introduced in previous publications. It describes general principles of intersegmental neuromuscular succession patterns to most efficiently generate specific movement intentions. The general hypothesis of this study is that these principles—if they really do indicate a *fundamental* basis for efficient movement generation—should also be found in *intramuscular* coordination and should be indicated by “longitudinal sequencing” between fibers according to the principles of the punctum fixum-punctum mobile model. Based on this general hypothesis an operationalized model was developed for the rectus femoris muscle (RF), to exemplarily scrutinize this hypothesis for the RF. Electromyography was performed for 14 healthy male participants by using two intramuscular fine wire electrodes in the RF (placed proximal and distal), three surface electrodes over the RF (placed proximal, middle, and distal), and two surface electrodes over the antagonists (m. biceps femoris and m. semitendinosus). Three movement tasks were measured: kicking movements; deceleration after sprints; and passively induced backward accelerations of the leg. The results suggest that proximal fibers can be activated *independently* from distal fibers within the RF. Further, it was shown that the hypothesized function of “intramuscular longitudinal sequencing” does exist during dynamic movements. According to the punctum fixum-punctum mobile model, the activation succession between fibers changes direction (from proximal to distal or inversely) depending on the intentional context. Thus, the results seem to support the general hypothesis for the RF and could be principally in line with the operationalized “inter-fiber to tendon interaction model”.

## Introduction

In previous publications we have already described the punctum fixum–punctum mobile model (compare [1–4] for details). This concept refers to the general principles of intersegmental sequencing of neuromuscular activation patterns for generating the *most efficient* kinematic output of multisegmental *co-directed* rotational movements.

The punctum fixum (Pfix) in the sense of this model is defined as the part of the body that is currently fixed at any rotational axis, and the punctum (or segmentum) mobile (Pmob) is defined as the free body part that shall be accelerated most efficiently. Depending on the specific kind of *intentional* movements, the main principles of this concept can be summarized as follows: (1) to generate the most effective *acceleration* of the Pmob, the intersegmental neuromuscular onset succession (INOS) has to run from Pfix to Pmob (whereas in *unpredictable* situations the succession runs inversely, from Pmob to Pfix) and (2) to generate the most effective *transfer of momentum* after the Pmob has been accelerated, the INOS has to run from Pmob back to Pfix. Within the framework of the individual context-specific intention, the variable that primarily determines the activation order to generate movements most efficiently is defined as the *relative distance from the rotational axis* in that model (compare [1–4] for details).

**Fig 1:**
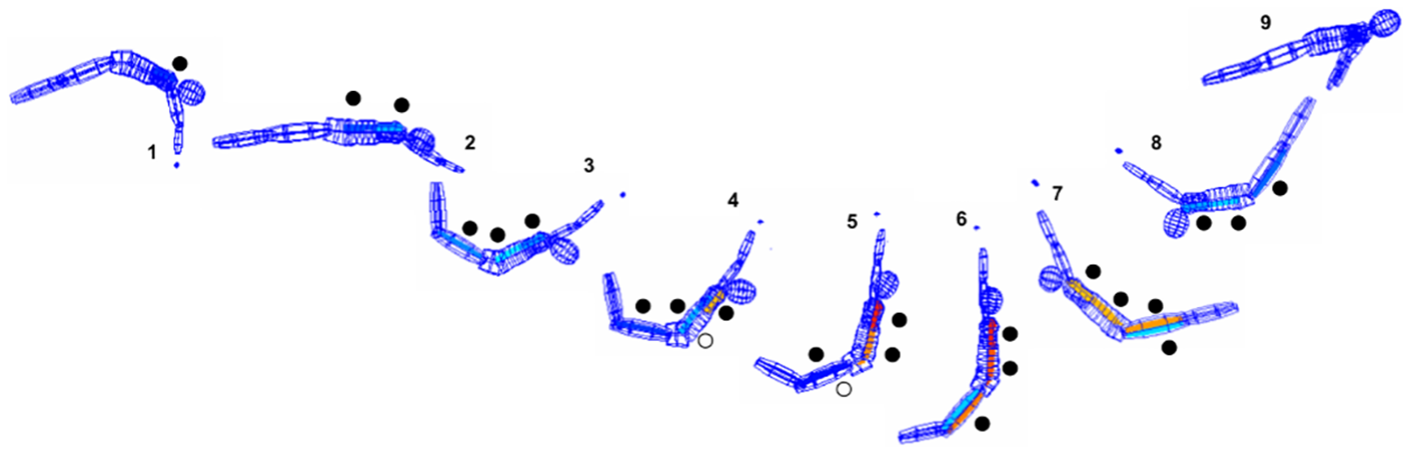
The sequence shows original kinematic and EMG data of a high-level gymnast performing the typical “whip-like” leg acceleration before dismount (compare [1, 3, 4]). The dots emphasize the overlapping IMS patterns between the posterior and anterior muscle chain, each running from Pfix to Pmob for an effective counter-acceleration of the segments (filled dots: muscles activated; empty dots: musculotendinous structures stretched). The positions 4–6 demonstrate the additional “longitudinal intersegmental catapult effect”: Whereas the proximal segments of the *agonist* chain have allready begun to spread, the more distal segments of the *antagonist* muscle chain are still activated. This longitudinal overlapping additionally enhances and extends the tension of the musculotendinous structures of the anterior chain to realize the whip-like acceleration most effectively. Immediately after the beginning of the acceleration of the Pmob the onsets of distal parts of the *antagonists* begin to run back towards the Pfix (beginning in position 7) to induce the subsequent transfer of momentum to the body. Acronyms: IMS: intertonic motor sequencing; Pfix: punctum fixum; Pmob: punctum mobile.

In the most effective “whip-like” accelerations, as often required in sports, we demonstrated that these principles result in successive stretch-shortening cycles running through the body [1–4]. To more specifically describe these characteristic interaction patterns based on the Pfix-Pmob model, we also called this: *intertonic motor sequencing* (IMS) [5]. These effects are even more supported by the *overlapping* nature of such longitudinal neuromuscular interaction patterns between the anterior and posterior muscle chain [3]. This effect could be also described as an “intersegmental longitudinal catapult effect” (compare Fig 1).

Logical considerations led to the following hypothesis: if the Pfix-Pmob model is indeed assumed to represent *fundamental principles* for efficient movement generation, then it should be expected that these functions should be found not only *between muscles* but also in the coordinative interaction of the functional units *within* a muscle. This hypothesis implicates the ability to intentionally induce a *longitudinal succession (sequencing)* between more proximal and more distal parts of a muscle, according to the context-specific principles of the Pfix-Pmob model.

The following indications could generally support our hypothesis. Similar to prior findings in animals [6–8], there are some indications that also several human spinalmotor muscles (e.g. the rectus femoris (RF), gastrocnemius and others) could be based on different functional units, not only in parallel arrangements, as widely known (e.g. deltoideus; biceps brachii or others), but also in longitudinal neuromuscular compartments [9–14]. Until now, however, no publication could provide any plausible explanation for the possible functional meaning of such longitudinal compartmentalization.

The aim of the present study is marked by two main objectives: (1) to develop an *operationalized model* based on the Pfix-Pmob model that is adapted to specific intramuscular structures; (2) to provide experimental evidence for the existence of “intramuscular longitudinal sequencing” as hypothesized. The operationalized model should be exemplarily adapted to the anatomical structures of the rectus femoris muscle (RF) by reasons that will be further explained in the Methods.

## Methods

### Ethics approval

All measurements were conducted according to the principles expressed in the Declaration of Helsinki and were undertaken with the understanding and written informed consent of each subject or their parents in case of juvenile participants. This includes consent for publication of their photograph, as outlined in the PLOS consent form for publication in a PLOS journal. The study was formally approved and permitted by the ethics commission of the University of Tübingen (project number: 645/2014BO1).

### Operationalization of the model

The RF was exemplarily chosen as the preferred muscle for this aim by the following reasons: (1) the RF is located superficially and therefore is easily accessible for FW-EMG and SEMG; (2) the RF is one of the longest muscles of the body, and this increases the chance to detect onset time differences between proximal and distal parts; (3) the muscle spans over two joints (hip and knee) and is therefore an ideal muscle to support efficient co-directed “whip-like” accelerations that the Pfix-Pmob model primarily refers to; (4) the muscle is innervated by neural parts being recruited from three different spinal segments (L2-L4). This anatomical fact could enable differentiated longitudinal activation of more proximal and more distal parts of the muscle; (5) in own pre-studies (using SEMG) we found different activation times over the proximal and distal parts of the RF, that were closely inter-coordinated within the intersegmental sequencing patterns according to the Pfix-Pmob model (see examples in S1 and S2 Videos); (6) in external studies there were indications that regional neuromuscular compartmentalization exists in the RF [11, 14].

Generally, the RF has a very complex 3D-structure (firstly described that way in 1995 [15]) that is marked by different architectural characteristics depending on the cross-section area over its length [9, 10], [15–23]. Here, we focus on those anatomical aspects that have direct functional consequences with respect to the development of the operationalized model, and that are also essential, to exactly retrace the specific positioning of the electrodes.

**Fig 2:**
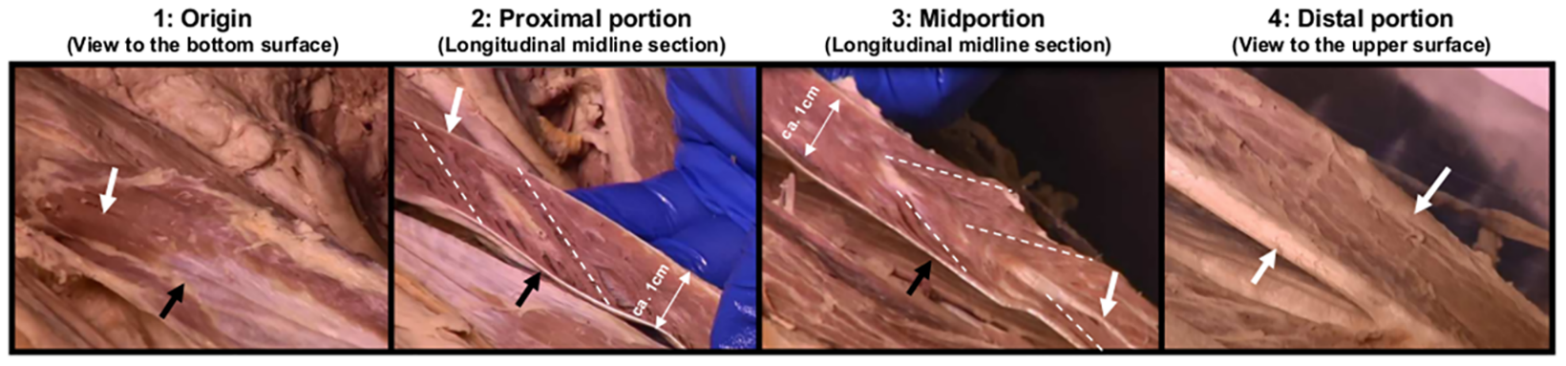
Anatomical structures of human RF (the left side of the pictures are directed to the origin). 1: Bottom surface turned to upside. View to the very proximal fibers (white arrow) inserting to the distal aponeurosis (black arrow) near the origin. 2: Longitudinal midline section through the proximal portion with unipennate fiber orientation (dotted lines) from the proximal aponeurosis (white arrow) to the distal aponeurosis (black arrow). 3: Midportion: The black arrow indicates the distal aponeurosis; the white arrow shows the course of the central cord of the proximal aponeurosis. The dotted lines indicate the radial orientation of the fibers in the more distal part. 4: Upper view to the distal insertion of the fibers into the u-shaped distal aponeurosis that finally transforms to the quadriceps tendon.

The RF is characterized by *very long aponeuroses* over its total length. These tendineous structures are divided into: (1) the proximal aponeurosis; (2) the distal aponeurosis; and (3) the structural transition of the distal aponeurosis into the “quadriceps tendon” that is continued by the “patella tendon”. The proximal aponeurosis is built by the tendineous structures of the direct head of origin (*caput rectum*), and proximally imposes as a superficial structure covering the proximal part of the RF. The muscle fibers run from this superficial part of the proximal aponeurosis to the distal aponeurosis that itself covers the back surface of the muscle. So, in this section the course of fibers is unipennate. In its further course the proximal aponeurosis changes its superficial structure to a more vertical and half-moon-shaped structure (built by the indirect head of origin [*caput reflexum*]) that runs more in the center of the muscle. In the distal half of the muscular length, this half-moon structure becomes smaller and runs more deeply and centrally (like a cord) in the muscle and is therefore also called “central aponeurosis.” In that section the muscle fibers run *radially* from this central cord to their insertions into the distal aponeurosis; in other words, in that muscular cross-section, the distal aponeurosis is more U-shaped clasping around the fibers (Fig 2).

**Fig 3:**
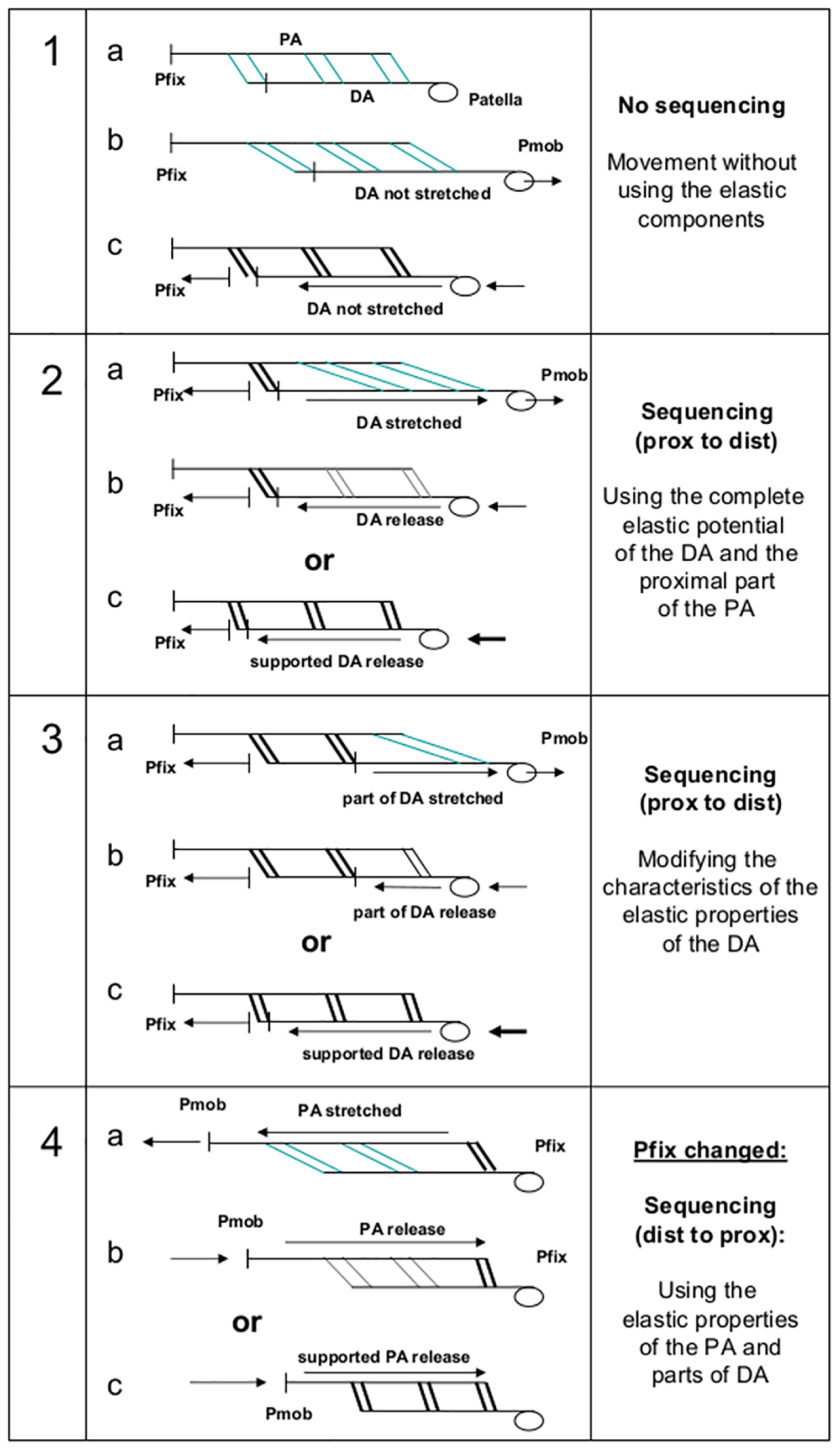
“Inter-fiber to tendon interaction model”: Fibers in bold are activated fibers (see text for further details). Acronyms: prox: proximal; dist: distal; Pfix: punctum fixum; Pmob: punctum mobile; PA: proximal aponeurosis; DA: distal aponeurosis.

Together, the *overlapping tendineous structures* of the proximal aponeurosis plus distal aponeurosis plus tendons measure approximately 70 to 80 cm in adults. This *high proportion of elastic components*, theoretically, allows for using these elastic structures efficiently like springs for storing and releasing energy during movements. Such spring-like muscle–tendon interactions have been frequently described in the literature. However, as also pointed out by Blemker et al. [21], the possible functional roles of the additional and summarized elastic components of all the involved *aponeuroses over their total length* are not adequately addressed in current muscle models. This aspect plays an important role in our operationalized model, as demonstrated in Fig 3.

Fig 3 shows a simplified functional scheme of the RF. For easier demonstration, the proximal and the distal aponeuroses are simply depicted as linear structures without giving attention to the anatomical fact that in the distal part of the RF, the proximal aponeurosis transforms to a central cord with radial fiber arrangement (see above). For further simplification in the drawings, only one head of origin is depicted (instead of the two heads), and only some proximal fibers, some distal fibers, and some fibers located in the midportion of the muscle (we call it here “middle fibers”) are demonstrated schematically, whereas in reality the fibers are arranged over the whole length of the muscle belly.

In case, fibers would be activated *synchroneously* over the whole length of the muscle (for example during a kick), the distal aponeurosis (DA) cannot be stretched effectively to store energy, and fibers would need to do the whole contractile work alone (1a, b, c in Fig 3). However, when specifically the *proximal* fibers (prox) are activated to get “stiff” during the muscle extension (2a in Fig 3), the DA will be stretched and can give the energy back during release (2b in Fig 3). In case the force of the release of the DA should *not* be *sufficient* for the intended movement (e.g. a strong kick), then the activation of the middle and distal fibers (dist) could additionally increase the resulting force during the release of the DA (2c in Fig 3). Additional stiffness of parts of the *middle fibers together with the proximal fibers* (3a in Fig 3) could even adjust the *kinetic characteristics* of the DA (to be “softer” or “harder”) by shortening the length of the “free part” of the DA. Furthermore, the middle fibers could influence the resulting force and timing during the “release phase” of the elastic structures by lower or higher additional contractile support in addition to the distal fibers (3c in Fig 3). According to the Pfix-Pmob model, example 4 shows possible conditions with the Pfix changed (when the foot is fixed on the floor), resulting in a *change* of the succession direction, using the elasticity of the proximal aponeurosis (PA) in that case. Such conditions could be found, for example, in the deceleration phase after a sprint when the foot contacts the floor.

The very specific architecture and the structural prerequisites of the RF muscle do, in fact, suggest the possibility of such functional myofascial interactions. The main prerequisite to support such differentiated interactions of the aforementioned model is the proof of clear evidence: (1) for the physiological ability of a *longitudinal differentiation* between muscle fiber activation and (2) for the coordinative ability to use *changing succession patterns* (prox to dist vs. dist to prox) that are adapted to the context-specific needs. These two aspects need to be proved within this study to support the defined hypothesis for the RF.

### Participants

Fourteen healthy male participants (height: 174 ± 7.50 cm; weight: 73 ± 8.92 kg) were recruited. None of them was a participant in one of our prior studies concerning the Pfix-Pmob model. Eleven of them were students of sport sciences at the University of Tübingen (aged 22–28 years). One participant was a 17-year-old boy and two other participants were athletic adults (aged 49 and 52 years).

### General description and procedure of the measurements

**Fig 4:**
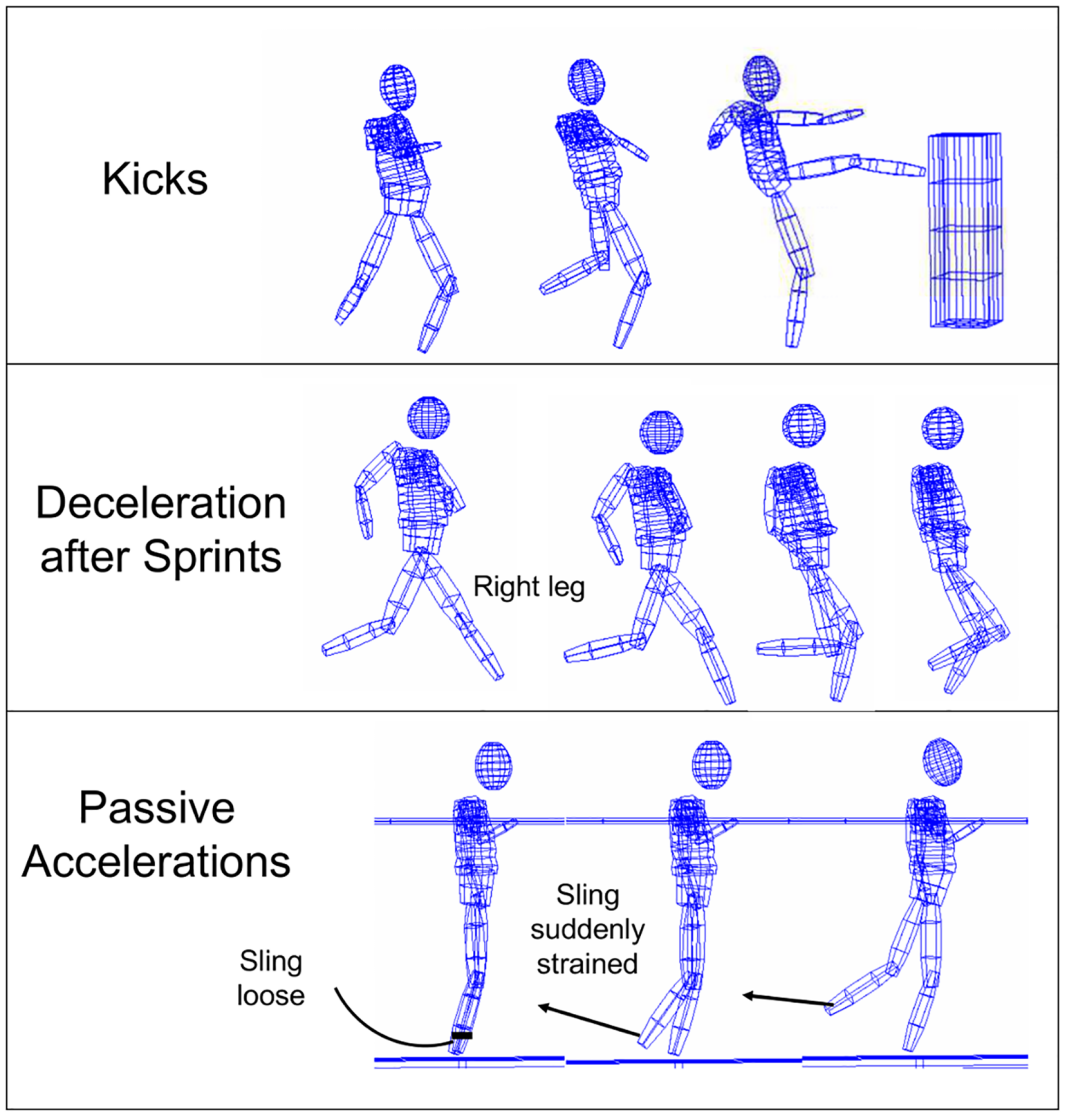
Examples of the three movement tasks that were measured. See text for details.

After careful preparation with FW and SEMG electrodes (details are provided later in the text) during the beginning of each participant's measurements maximum voluntary contraction tests (MVC) in different positions were recorded. These tests were performed to obtain a reference for the EMG signal quality and to estimate the individual characteristics of all the derivations in each participant. After the MVC measurements were finished, the participants had to perform the main measurements with a series of three movement tasks (see Fig 4).

Prior to the measurements of each of the tasks, a series of initial practice tasks were performed by the participants so they could become used to the specific requirements. Each measurement of the “kicks” and the “passive accelerations” included six repetitions with adequate breaks for regeneration and sufficient mental concentration. However, the “sprints” were performed only two times to avoid injuries caused by neuromuscular fatigue.

The three specific movement tasks were chosen for the study for the following reason: *all* of the movements consist of co-directed rotational components of upper and lower leg segments that include a combined dynamic activation of the RF for hip flexion and knee extension. So, these conditions are in accordance with our prior studies that resulted in the described Pfix-Pmob model for the same general movement characteristics (co-directed accelerating multi-segmental movements, with changing Pfix). The *different characteristics* between the three chosen movement conditions in the present study are as follows: the “kicks” are predictable, actively induced *positive* accelerations (with the *hip as Pfix*); the “deceleration steps after sprints” are also predictable and actively induced but are *negative* accelerations with additionally *inverted Pfix-Pmob conditions* (pfix = foot); and the “passive accelerations” are *unpredictable*, *passively induced*, *and actively stopped negative accelerations* (again with the *hip as Pfix*). One kind of movement that is described in the Pfix-Pmob principles (see Introduction) could not be specifically addressed within this study. It was any kind of movement that includes a *transfer of momentum* from Pmob to Pfix. Such a movement is difficult to create specifically, regarding the RF, and was therefore not included in the measurements.

The movements were to be performed as follows: the kicks should be performed in the manner of a kickboxing movement and should be performed as powerfully as possible against soft upholstery at chest height. This movement was chosen because the leg has a long active acceleration and, because of the more *horizontal* trajectory of the movement, is less affected by external forces like gravity, as is the case for “normal” kicks, which are primarily performed in the sagittal plane. The kickboxing movement is characterized by a hip flexion in the beginning of the movement (while the knee gets even more bent) and a supportive knee extension at the end of the kick. So, these characteristics fulfill the typical conditions of efficient “whip-like” accelerations of the Pmob as they are related to the Pfix-Pmob model. The “standardization” of the movements was limited to describe and demonstrate the general movement. Each participant should try to realize the skill, as he feels is best to do, to support a kind of movement that is *as natural and intuitive* as possible with the most powerful movement output. According to the definition of the Pfix-Pmob model (and as also carried out in our prior studies), no standardization other than the *maximum intention* for highest individual output performance was set as priority to represent the individual natural coordination patterns best without being inhibited by strict external preconditions. Even though this low grade of standardization may result in a higher inter- and intra-individual variability of the kinematic output, this approach was consciously chosen as a *fundamental prerequisit* to reveal the *most functional* activation patterns.

The “deceleration steps” were naturally performed by the participants to efficiently slow their speed after a sprinting start. Again, there was no additional advice other than to start as powerfully as possible and to slow down in time to avoid running into the equipment at the edge of the runway with a total length of about 13 m. This included about three to four powerful steps of a fast sprinting start from a “sprint block”, directly followed by a strong deceleration. Each first “deceleration step” of the right leg was chosen for analysis. The beginning of the deceleration phase was detected by video analysis, marked by that obvious change of the relation between center of gravity (COG) position and foot position during heel strike in a *body position slightly leaned to backward* (compare Fig 4) in contrast to the preceding steps during the sprinting start, marked by a *body position leaned far to forward*.

The “passively induced accelerations” were realized in a position with the left leg standing on a 20-cm-high stable surface and the right leg hanging to the side without touching the floor. To keep their balance, the participants were fixed with their left arm and the right arm was also hanging next to the body. A non-elastic sling was fixed at the right ankle. In the beginning and between the trials, the sling was without any tension. In unpredictable moments the sling was suddenly pulled backward over a distance of approximately 30 cm. This induced an initial passive knee flexion, followed by a passive hip extension (compare Fig 4). During the moment when the participant feels the pull, he should try to immediately stop the passive movement of the leg. Attention was given to the fact that the right leg was always *completely relaxed* before and between the trials.

During all the measurements, additional video data were captured and synchronized with the EMG data by an optical and analog trigger signal (see later in the text). Between each of the measurements the participants took a break for approximately 1 minute.

### Data acquisition and post-processing

Wireless surface electromyography (SEMG) and fine wire electromyography (FW-EMG) were captured synchronously at 3000 Hz by the 8-channel system Telemyo 2400T (Noraxon, Scottsdale, AZ, USA). SEMG uses electrodes that are placed on the skin over the belly of the muscle that will be measured. FW-EMG is used for intramuscular EMG measurements by fine wires (FW) that are applied to the muscle by a needle and kept in the muscle for the duration of all measurement procedures. The sterile sets of needles are each prepared with a pair of integrated fine wires (specification: 0.50x50mm, 10 CM wire; SD59-000-250, Spes Medica, Battipaglia, Italy). The insulation of the tips of the FWs is dismantled over 5 mm and slightly spread and hooked for easier introduction and for anchoring within the muscle tissue while the needle is retracted after correct placement.

Using one channel of the EMG system for the input of the trigger signal (see later in the text), seven channels were available for EMG derivations of each the participant’s right thigh. Three pairs of SEMG electrodes were fixed *over* the RF and two FW-EMG electrodes were placed *in* the RF. Another two pairs of SEMG electrodes were placed over the muscle belly of the main antagonists (biceps femoris muscle [BF] and the semitendinosus muscle [ST]). The EMG preparation was in accordance with the standards of the International Society of Electrophysiology and Kinesiology (ISEK-Standards; [24]).

#### Placement of the electrodes

FW: The application of the *FW electrodes* was performed in accordance to the rules and hygienic principles of deep muscle punctures [25]. The placement of the electrodes was adjusted in relation to the bone landmarks and directed to specific intramuscular positions as they were detected by continuous ultrasound control during the entire procedure of the electrode application. To ensure a best possible distinction of the results only *two* FW electrodes were applied; one in the proximal and a second in the distal part of RF. This decision was caused by the fact that in the complex 3D-structured midportion of the muscle the origins and insertions of singular fibers cannot be detected in detail by 2D-ultrasound imaging. So, it is *not* possible to precisely differ between proximal, middle and distal fibers *in the midportion* of the muscle. Therefore, using *more than two* FW electrodes would have been *additionally* enhanced the variability of the FW results that are caused for example by unknown types of the punctured fibers (fast or slow twitch) and the unknown distances between FW positions and innervation zones. Because of the complex structure of the RF, it is very difficult to clearly identify the innervation zones in human RF [26, 27]. Therefore, we did not try to detect the innervation zones in this study.

**Fig 5:**
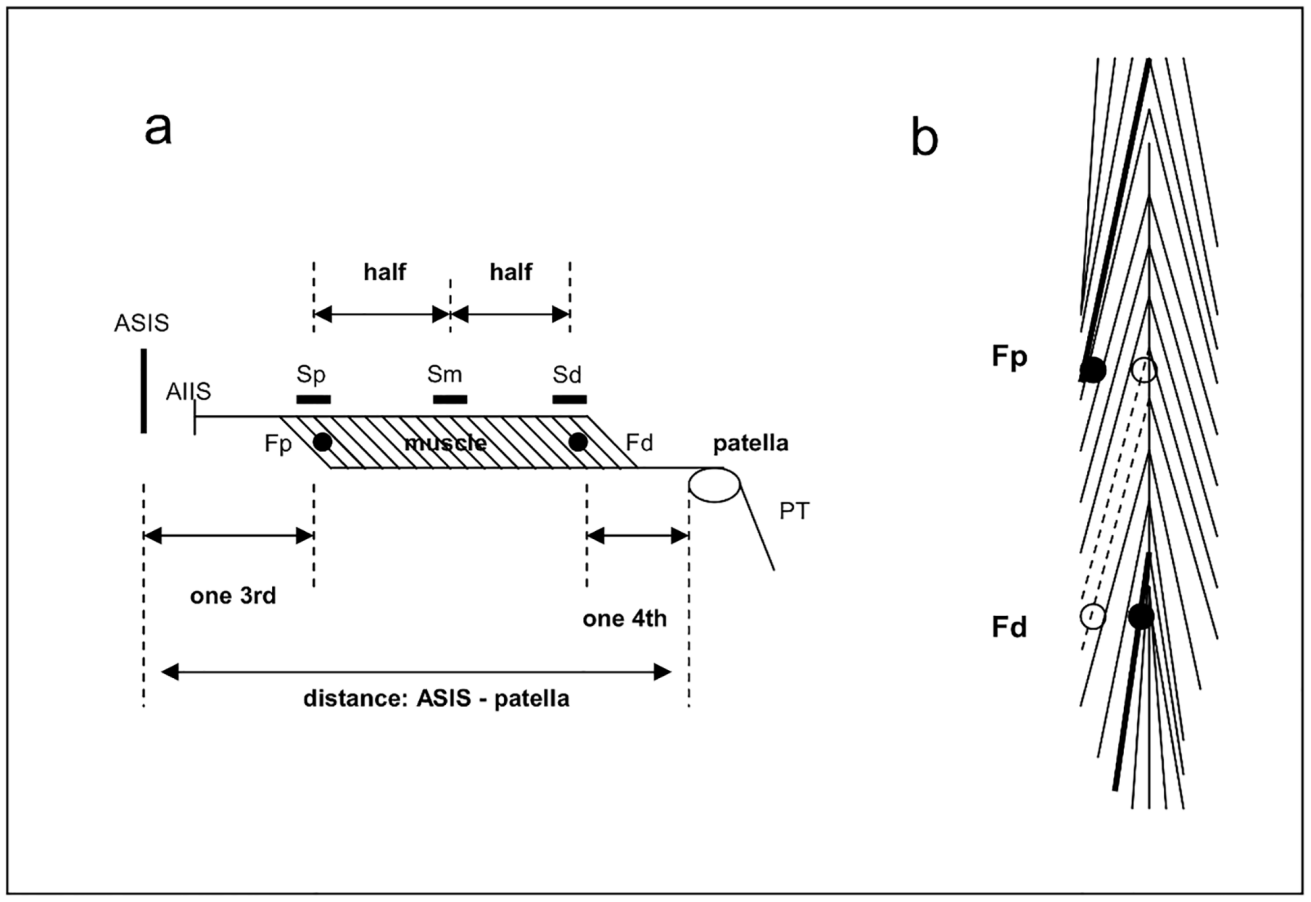
Electrodes positions. Please note that for easier depiction the proximal and the distal aponeuroses are simply depicted as linear structures without giving attention to the fact that in the distal part of the RF the proximal aponeurosis transforms to the central cord with radial fiber arrangement. Further, only one head (instead of the two heads) is depicted here and fibers are longer with smaller pennation angles than depicted in drawing a. a) Placement of the bipolar SEMG electrodes and FW electrodes from lateral perspective. b) Placement of the FW-electrodes in the frontal plane. The *filled circles* indicate the FW positions, as directed by ultrasound control (depicted as thick lines). The *empty circles* demonstrate that a more medially placed Fp and a more laterally placed Fd in the same cross-section area could meet nearly the same fibers (depicted as dotted lines). See text for further details. Acronyms: ASIS: anterior superior iliac spine; AIIS: anterior inferior iliac spine; PT: patella tendon; Fp: fine wire, proximal derivation; Fd: fine wire, distal derivation; Sp: SEMG, proximal derivation; Sm: SEMG middle derivation; Sd: SEMG distal derivation.

The *proximal electrode position* was one-third the distance from the “anterior superior iliac spine” (ASIS) to the upper edge of the patella (Fig 5). This FW electrode was placed *eccentrically* in the lateral and deep third of the unipennate part of fibers running from the proximal aponeurosis to the distal aponeurosis (see Fig 5). This specific eccentric position was chosen for two reasons. First, the placement in the lateral third should ensure the meeting of the long fibers of the *unipennate* lateral part of the muscle. Second, the placement in the deep third should ensure the meeting of the more distal parts of these fibers (inserting in the distal aponeurosis), whose origin is located more proximal. This specific placement of the proximal FW electrode should ensure the measurement of fibers located *most proximal* in the muscle.

The *second FW electrode* was placed in the distal RF, one-quarter proximal to the distance between the upper edge of the patella and the ASIS. In contrast to the proximal electrodes position, the distal FW electrode was placed near *the center* of the muscle. This specific placement was chosen because a position *near* the center should ensure placing the electrode as closely as possible to the *origin* of fibers, running from the central aponeurosis in a radial shape to the most distal part of the muscle (inserting in the U-shaped distal aponeurosis). Placement of the electrode more *eccentrically* (as used in the proximal FWs) would meet the more distal parts of those fibers coming from a more proximal origin (compare explanation in Fig 5).

SEMG: Using additional SEMG served to underline the FW results by an independent second method. In contrast to the decision to use only *two* FW derivations, in the SEMG additionally a *third pair* of electrodes could be placed exactly between the proximal and distal derivations (see Fig 5) to better detect progressive sequencing patterns over the whole muscle length. This middle derivation additionally represents the standardized position of SEMG measurements over the RF as defined by the SENIAM standards (SENIAM: surface EMG for non-invasive assessment of muscles, [28]) and therefore served as a reference concerning standard SEMG studies. In completion to the regionally *localised* and RF specific FW signals, SEMG allows a more *global sight* over a bigger volume of the thigh (even if crosstalk influences cannot be excluded). This parallel approach with FW and SEMG allowed it to use the specific advantages of both techniques, partly compensating for the disadvantages of using only *one* of them.

*The SEMG electrodes* were placed as follows: After shaving and fine grinding the skin area with mild sandpaper, three pairs of gel-covered silver / silver chloride (AG/AgCL) disc electrodes (H92SG, Kendall Arbo, Tyco Healthcare GmbH, Neustadt/Donau, Germany; radius 1 cm) were placed with an inter-electrode distance of 2 cm in alignment with the fiber direction. A reference electrode was attached to the ASIS. Electrode skin impedance was accepted at a level of <5 kOhm. The proximal and distal pairs of electrodes were located directly over the proximal and distal FW electrode positions. Finally, the SEMG electrodes over the BF and ST were placed at the standardized positions over the middle of the muscle belly.

All electrodes, amplifiers, and wires were fixed on the skin with tape in the maximum stretched position of the RF to prevent tension or dislocation during maximum movement amplitudes and to avoid movement artifacts. Finally, a specifically prepared elastic body suit was put on and carefully adjusted to additionally affix the transmitters, amplifiers, and wires tightly to the body, without disturbing the participants’ movements.

#### Data synchronisation

The derivations of all FW-EMG and SEMG data were synchronously detected during the capturing process using the wire-based entrances to the same data logger before their common transmission to the stationary computer system, where all data were stored for further processing. A receiver for detection of the EMG trigger signal was connected with the EMG data logger and was also affixed at the body. This signal was visualized on a separate channel in the EMG recording. The video system (Panasonic 3CCD, NV-GS230, Kadoma, Japan) was triggered by a LED (light emitting diode) signal that was synchronized with the EMG trigger signal. Based on the trigger signals, the video data and EMG data could be synchronized within the EMG software Myoresearch (version 1.06.60; Noraxon, Scottsdale, AZ, USA).

#### Data detection and processing

The following standards of EMG detection were used as specified by the manufacturer: input impedance, >100 MOhm; CMR (common mode rejection ratio) >100 dB; and SNR (signal-to-noise ratio), baseline noise <1 μV RMS (root mean square). The raw EMG signals were bandpass-filtered (10–500 Hz) and sampled at 3000 Hz, analogue-digital (AD)-converted (12 bit), and stored for further processing in the stationary computer system. For further analysis, the EMG data were full-wave-rectified and smoothed over a constant time window of 50 ms (root mean square [RMS]) by using Myoresearch software (version 1.06.60; Noraxon, Scottsdale, AZ, USA).

#### Onset detection procedure

**Fig 6:**
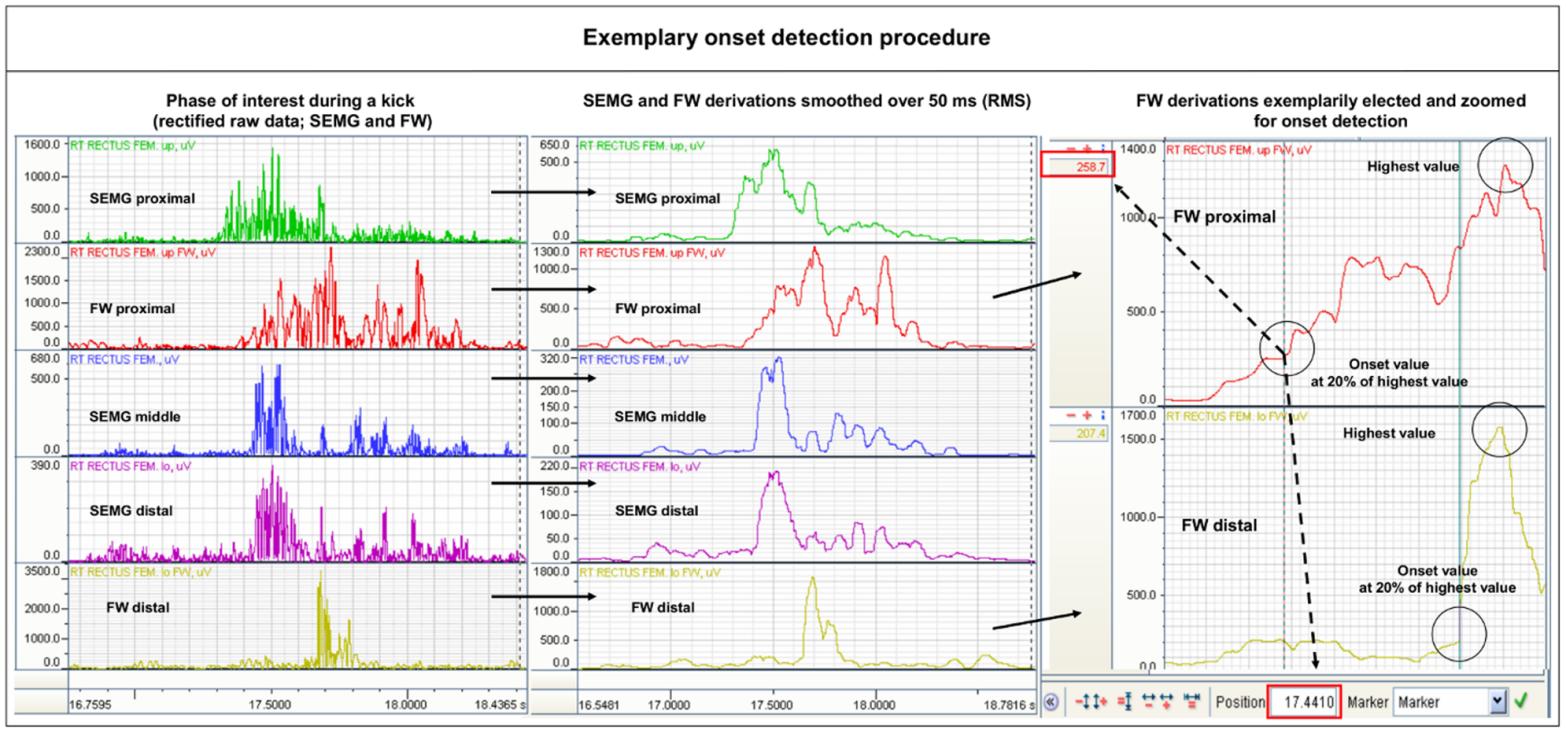
Manual onset detection procedure. Left: rectified raw data of fine wire EMG (FW) and surface EMG (SEMG) traces during a kick; middle: smoothed data traces; right: FW traces are elected and zoomed to detect the peak amplitude value during the phase of interest (each highlighted by the upper circle). This value is divided by 5 (20% threshold). The exact time of the frame that firstly reaches or exceeds this calculated threshold value (highlighted by the marker lines and the lower circles) represents the onset. In this example the 20% threshold of *proximal FW derivation* is crossed at 258.7 μV, resulting in an onset value of 17.4410 s (indicated by the dotted arrows). Same procedure is also used for distal FW and the SEMG traces (not depicted in this figure). Based on the detected onset times, onset differences between the derivations were calculated for statistical analysis.

For conditions of *highly dynamic natural movement tasks* with different levels of pre-activation as they were investigated in this study, the used method for *onset detection* seemed to be most suitable for generating valid data. According to our prior publications concerning the Pfix-Pmob model, the data analysis for all movements was primarily focused on the *sequencing patterns* of the EMG data. They were defined as the patterns of the temporal onset succession between the different SEMG and FW derivations. The onset threshold was set at 20% of the peak value of the movement sequence of interest. This threshold definition referencing varying amplitudes of single trials is also recommended in the literature for onset analyses of singular acyclic movement phases [29]. The onset detection procedure was carried out manually for all trials. The onset of each derivation was defined as the first frame that reaches or crosses the onset threshold in processed data traces (compare Fig 6). The onset value was only accepted if it remained over the threshold until the peak of the signal was reached.

To scrutinize if there exists a similar “*intra*muscular longitudinal catapult effect” during the “kicks” (whip-like acceleration) as it was found by the overlapping agonist/antagonists interaction in *inter*muscular coordination patterns during whip-like movements (see Introduction; Fig 1), the temporal relationships between the agonist’s *onsets* and the antagonist’s *offsets* (BF, ST) during the kicks had to be calculated. In accordance to the *onset* detection, the threshold for the *offset* detection was also set at 20% of the last peak during the phase of interest.

Inter-rater reliability of the onset detection method was > 0.99 (ICC 3, 1; n = 513; outliers excluded). The mean inter-rater deviation was 0.0002 s (SD: 0.015). Outliers with detection differences > 0.099 s have been found in 3.2% of the cases.

### Statistical analysis

To get balanced numbers of data per subject, for *kicks* and *passively induced movements* each the three trials 2–4 (midportion of the measurements) were chosen for further statistical analysis. In cases of artifacts or other reasons disabling clear onset detection in *any trace* (for example in case a clear peak could not be defined during the phase of interest in a trace), trials were *completely removed* from further analysis and substituted by the next trial (in the following order: 5, 6, 1). Caused by the fact that *decelerations after sprints* were performed only two times per participant (see above), in this case all evaluable trials were selected according to the above mentioned criteria.

All data that were included in the analysis were described by calculating the total mean and standard deviation (SD) of the onset times. Effect sizes were calculated using Cohen’s *d* [*d* = (mean *x*–mean *y*)/mean SD], with values of 0.2 < *d* < 0.5 defined as small effects, 0.5 < *d* < 0.8 as moderate effects, and > 0.8 as large effects [30]. To give additional insight into the singular counts of demonstrated patterns (prox to dist or dist to prox), the proportional representation of these patterns (in %) are provided. In this case the patterns were separately counted for each trial as a pure *order* of the intramuscular onset succession, *independent* of the time differences between the derivations.

For inferential analyses, we applied a linear mixed model for overall mean estimation to obtain an unbiased test statistic. This decision was based on the fact that the design includes multiple measurements for each test person and for each activity. Hence, we do not have independent observations, and standard inferential tests may lead to biased test statistics. Because we assume that the measurements for a test person are dependent, we modeled them as random factor. In the second stage, we compared the mean difference of the three movement tasks (kicks; deceleration; passively induced accelerations). Again, a linear mixed model with activities as the independent variable was applied. Furthermore, movement task and test person applied were modeled as random factors because we assume that the intra-person and intra-task measurements are not independent. Finally, a post hoc pairwise comparison of activities with Bonferroni correction was applied. Residual analysis was performed to test the normality assumption of the linear mixed models. The alpha level was set at p = 0.05. SPSS V21 (IBM, Armonk, NY, USA) was used to conduct the statistical analyses.

To give an estimation of the reproducibility of the measurements, additional replication tests were conducted. For this purpose one of the participants was tested in a second session (post-test), three weeks after the main measurements (same setup and conditions, as defined). Kickbox movements were chosen for the analysis because this task represents activation patterns of all muscles (including the antagonists) and is marked by a clearly detectable “moment of impact”, the data could be synchronized to. Values of each 2000 frames (0.66 s) prior to impact were included to the analysis. Correlation coefficients (Pearson) for all EMG derivations were calculated within each session and between the sessions. Fisher’s z-transformation procedure (and re-transformation) was used for calculating averaged values.

## Results

### Replication tests

Averaged r-values of correlation coefficients between the kicks within the *pre-test* (n = 15 intra-session comparisons) were found for SEMG derivations with: Sp: > 0.73, Sm: > 0.72, Sd: > 0.72, BF: > 0.73, ST: > 0.75. FW derivations: Fp: > 0.48; Fd: > 0.90. Averaged r-values within the *post-test* (n = 10 intra-session comparisons): SEMG derivations: Sp: > 0.72, Sm: > 0.67, Sd: > 0.74; BF: > 0.63, ST: > 0.53. FW derivations: Fp: > 0.63; Fd: > 0.94. Averaged r-values of the *pre-post analysis* (n = 30 pre-post comparisons): SEMG derivations: Sp: > 0.70, Sm: > 0.70, Sd: > 0.69; BF: > 0.72, ST: > 0.63. FW derivations: Fp: > 0.42; Fd: > 0.91. Averaged correlation coefficients for all intra-test comparisons (pre-pre and post-post) were found with: r > 0.70 for SEMG and r > 0.81 for FW. Averaged correlation coefficients for all the inter-test comparisons (pre-post) were found with: r > 0.70 for SEMG and r > 0.77 for FW. Compare S3 Table for all detailed r-values of the original data sources.

### Sequencing patterns within the FW and SEMG derivations

**Fig 7:**
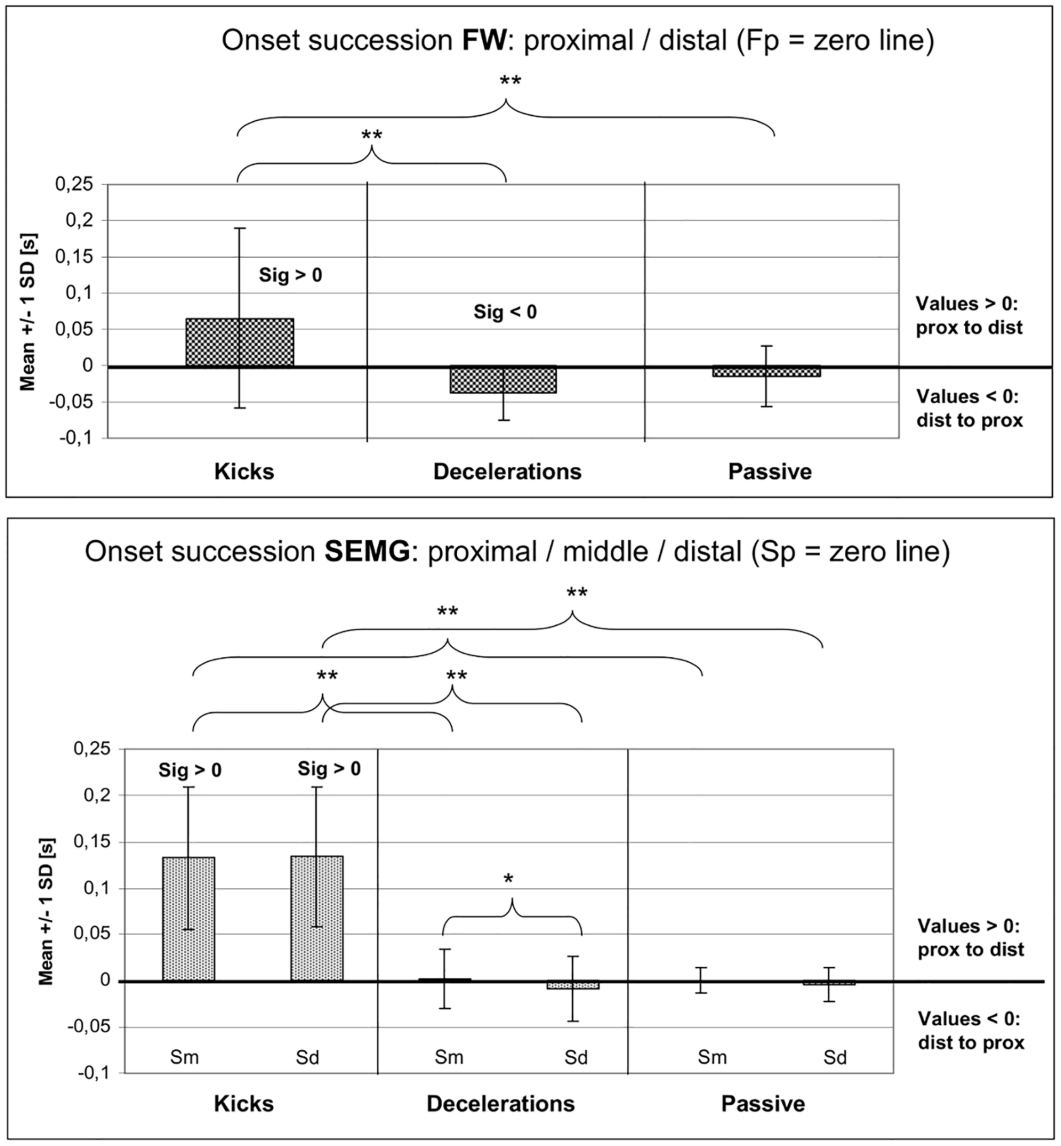
Onset differences (mean +/- 1 SD) between fine wire (FW) derivations (upper graph) and surface EMG (SEMG) derivations (lower graph) for the three movement tasks (kicks: n = 38; decelerations: n = 17; passive accelerations: n = 41). The zero line is each referenced to the proximal derivations (Fp in FW; Sp in SEMG data). The bars represent the time differences between proximal (= 0) and middle or distal derivations. Sig>0 (or Sig<0) = significantly different from zero; * = significant differences with p < 0.05. ** = significant differences with p < 0.01. Acronyms: prox: proximal; dist: distal; Fp: fine wire proximal derivation; Sp: SEMG proximal derivation. Sm: SEMG middle derivation; Sd: SEMG distal derivation; SD: standard deviation.

In the *FW measurements* clear temporal differences between proximal and distal onsets could be observed (upper graph of Fig 7; see also S1 Table of supporting information). The patterns of the *kicks* were marked by significant earlier onsets of the proximal versus the distal derivation (p = 0.046, with 69.2% of all cases representing the pattern prox to dist), even though the variability between proximal and distal FW onsets during the kicks was very high with values up to 0.47 s [prox-dist] and -0.1 s [dist-prox].

In contrast to this, during the *deceleration after sprints* the FW patterns were *mainly opposite of that* (with 88.2% of all trials showing the pattern dist to prox; p = 0.004), with remarkable shorter latencies between the onsets than during the kicks. In the *passive accelerations* the FW patterns prox to dist and dist to prox were more balanced and did not differ significantly from zero (p = 0.137). The onset differences of the FW derivations in comparison *between* the movement tasks were also highly significant in kicks vs. decelerations (p = 0.004) and kicks vs. passively induced movements (p = 0.015). Decelerations and passively induced movements, however, did not differ significantly concerning the FW onset patterns (p = 1.000).

In the *SEMG data* (lower graph of Fig 7) the onset differences were similar to the results of the FW. The patterns of the *kicks* were again marked by significant earlier onsets of the proximal versus the *middle* and *distal* derivation (with each p < 0.001). In this case, the percentages of the patterns prox to middle and prox to dist, as well, were each *100%*, with onset differences up to 0.29 s. Similar to the FW data, the patterns during the kicks differed significantly from decelerations and passive movement tasks (all with p < 0.001), whereas decelerations and passive movements did not differ significantly. In decelerations and passive movements latencies between proximal and distal derivations were again shorter than during the kicks and patterns prox to dist vs. dist to prox were widely balanced (with 58.8% in decelerations [p = 0.632] and 50% in passive movements [p = 0.197]). Looking specifically at the onsets of the *middle vs*. *distal derivation*, only minimal differences could be detected. Only during *deceleration movements* distal onsets were significantly earlier than the onsets over the middle derivation (in 72.5% of the cases [p = 0.02]).

### Onset differences between SEMG and FW derivations over the same cross section areas

**Fig 8:**
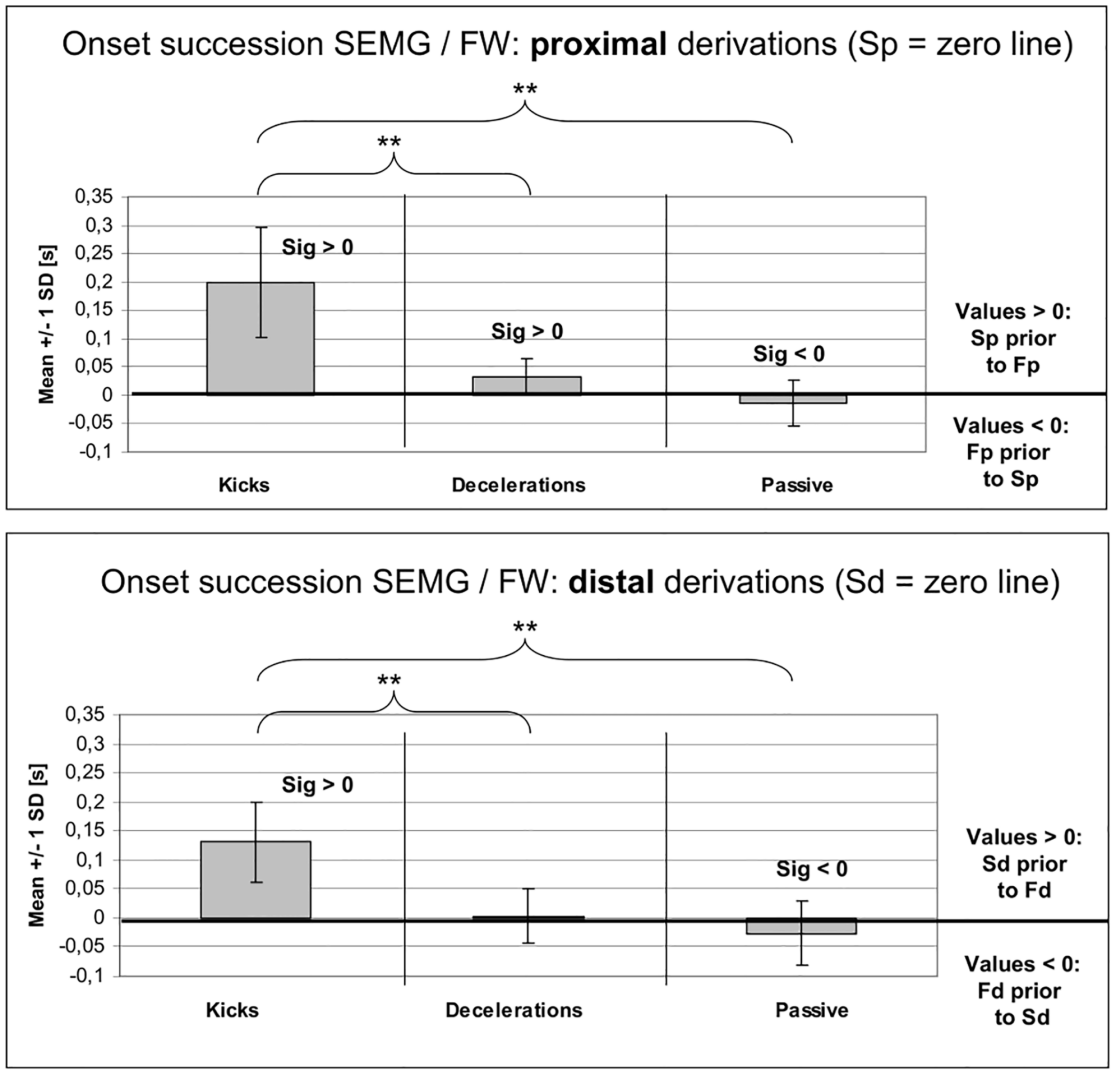
Onset differences (mean +/- 1 SD) between surface EMG (SEMG) and fine wire (FW) derivations over the same cross section areas for the three movement tasks (kicks: n = 38; decelerations: n = 17; passive accelerations: n = 41). Upper graph: derivations over the proximal cross section area; lower graph: derivations over the distal cross section area. The zero line is each referenced to the SEMG derivations (upper graph Sp; lower graph Sd). Sig>0 (or Sig<0) = significantly different from zero; ** = significant differences with p <0.01. Acronyms: Fp: fine wire proximal derivation; Sp: SEMG proximal derivation; Fd: fine wire distal derivation; Sd: SEMG distal derivation; SD: standard deviation.

Fig 8 shows that during the *kicks* the SEMG onsets were significantly *earlier* than the FW onsets over both cross section areas (proximal in 97.4% and distal in 100% of all trials; both with p < 0.001). In *deceleration movements* the SEMG onsets occurred, again, mostly *prior* to the FW derivations (proximal in 88.2% [p = 0.003], distal in 64.7% [p = 0.86]), but with considerably shorter delays compared to the kicks. In the *passive accelerations*, however, the onsets of the SEMG derivations were significantly *later* than the ones of the FW derivations (proximal in 57.5% [p = 0.05], distal in 67.5% [p = 0.036]). S1 Table shows the complete statistical data. S2 Table shows the original data over all participants.

### Synopsis of context-specific sequencing over all derivations (FW and SEMG)

**Fig 9:**
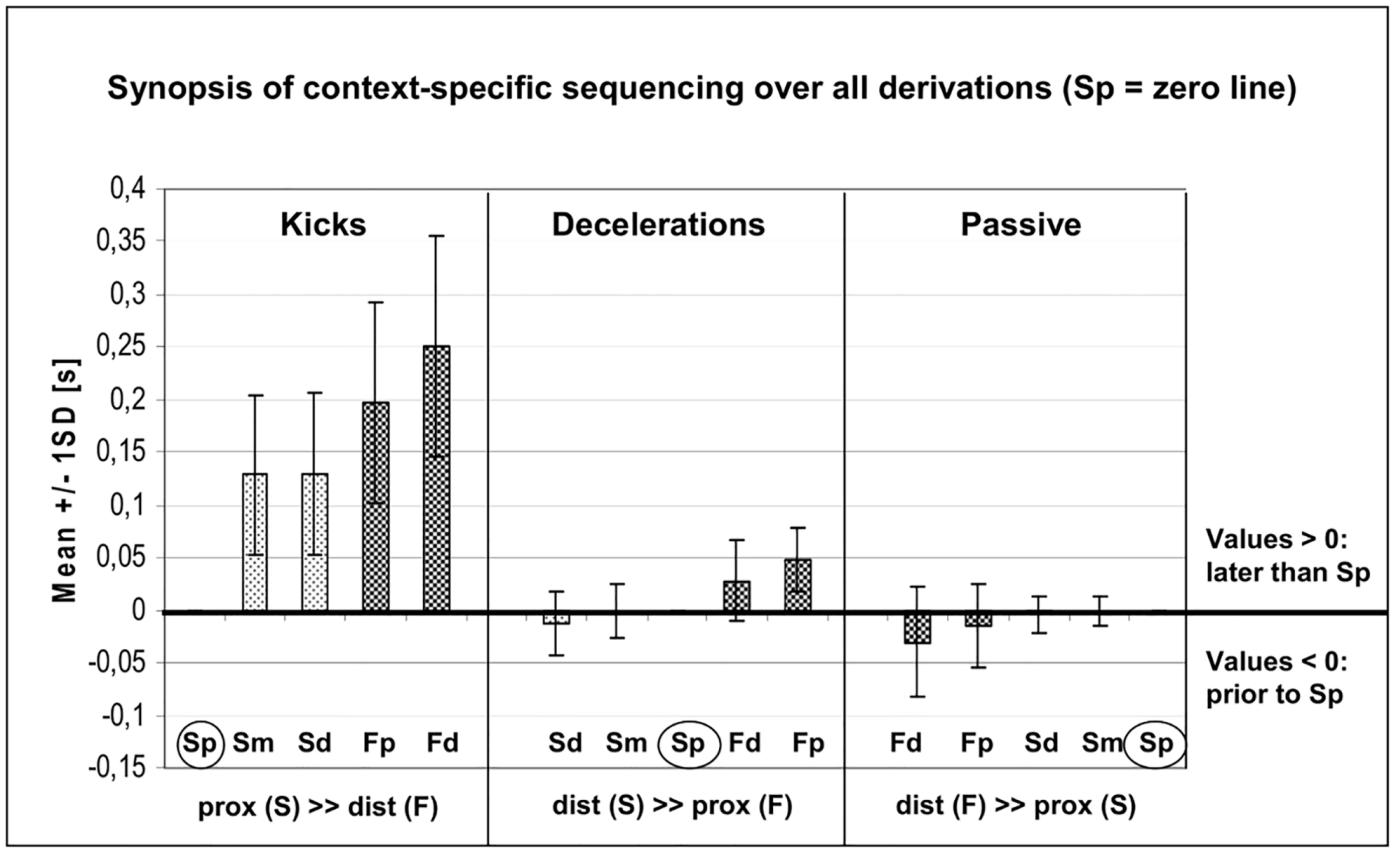
Agonist’s sequencing patterns over all the measured movement tasks (mean ± SD for SEMG and FW). The darker bars each represent the FW derivations. The zero line of all graphs is referenced to the onset values of Sp (highlighted in circles). The bottom line of the figure summarizes the mean succession patterns (prox to dist or dist to prox); the letters in parentheses indicate, whether the SEMG (S) or FW (F) derivations begin the sequencing. Acronyms: prox: proximal; dist: distal; F: fine wire; Fp: fine wire proximal derivation; Fd: fine wire distal derivation; S: SEMG; Sp: SEMG proximal derivation; Sm: SEMG middle derivation; Sd: SEMG distal derivation.

Summarized, Fig 9 demonstrates the sequencing patterns *over all derivations*, referenced on the Sp onsets over all movement tasks. The graphs integrate the same results as outlined above, but give a better overview concerning the differences *within and between* the EMG methods, referenced to the context-specific conditions of the three movement tasks.

### Agonist-antagonist interaction during the kicks

**Fig 10:**
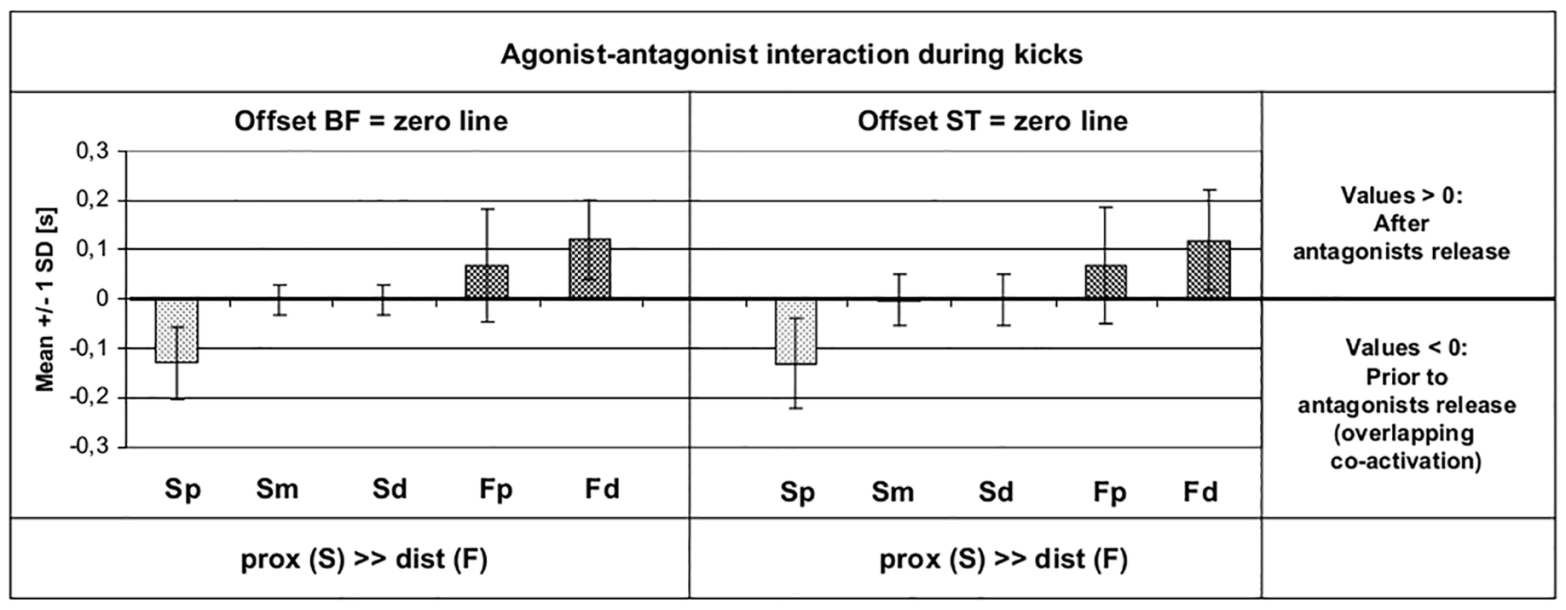
Temporal interaction between *agonist* onsets and *antagonist’s* offsets during the sequencing of *kicks* (mean ± SD for SEMG and FW; n = 38). The darker bars each represent the FW derivations. The zero line is referenced here to the *offsets* of BF (left) and ST (right). Negative onset values (below zero line) indicate that these onsets already begin when the antagonist is still co-activated (overlapping of antagonist and agonist activations). Positive values show onsets that begin *after* release of the antagonist. Acronyms: BF: biceps femoris muscle; ST: semitendinosus muscle; prox: proximal; dist: distal; F: fine wire; Fp: fine wire proximal derivation; Fd: fine wire distal derivation; S: SEMG; Sp: SEMG proximal derivation; Sm: SEMG middle derivation; Sd: SEMG distal derivation.

Fig 10 additionally focuses on the *intra*-segmental agonist-antagonist interaction over all derivations during the kicks (whip-like acceleration), to scrutinize if there exists a similar “*intra*muscular (btw. intrasegmental) catapult effect” as it was found by the overlapping agonist/antagonists interaction in *inter*muscular coordination patterns during whip-like accelerations of the Pmob (see Introduction; Fig 1).

The figure makes clear that Sp onsets *overlap* with the antagonist’s activation. This co-activation with BF was found in 100% of all kicks (p < 0.001), with ST in 92.3% of all the kicks (p < 0.001). The onsets of the *middle and distal* SEMG derivations, however, were widely *synchronous* with the offsets of the antagonists; the *proximal FW* onsets began partly earlier, partly later than antagonists offsets; the *distal FW* onsets mostly began *after* the antagonist’s offsets (in 89.7% after BF; in 94.9% after ST [each with p < 0.001]). So, the findings show significant *co-activation* of only the *proximal parts* of the agonist, whereas the *middle and distal parts* of the agonist are mostly activated *together with*, *or after* the antagonist’s release.

### Supplementary findings

In small leg movements (e.g. during preparation between the measurements), *isolated activations* of proximal or distal activity in FW and SEMG were frequently apparent *in all participants* (compare also S6 Video). In bigger steps (e.g. before or after the measurements) “prox-to-dist patterns” have been typically observed during the *swing phase*, “dist-to-prox patterns” with the transition to the *stance phase* (compare S7 Video). These observations will be further adressed in the discussion section.

## Discussion

### Summary of the main findings

The main findings of the study can be summarized as follows. The results provide evidence for the human ability to differentiate between more proximal and more distal parts of the RF muscle. This ability of longitudinal differentiation can be used in a manner of functional and context-specific “longitudinal sequencing” from prox to dist or from dist to prox. During *intentional accelerations* (kicks) the onsets primarily run from Pfix (hip) to Pmob (foot). Under conditions of *intentional accelerations* with a *change of the Pfix* (during deceleration movements), the onset patterns clearly tend to *change their direction* (from dist to prox), *keeping the succession* from Pfix to Pmob. In *unpredictable*, passively induced accelerations of the Pmob, the onsets also clearly tend to *change their direction* (from dist to prox) and *also change their succession* (from Pmob to Pfix).

### Aspects concerning the results of the replication tests

The replication tests showed widely similar mean correlation coefficients in intra-session comparisons (r > 0.70 for SEMG, r > 0.81 for FW) as they were found in inter-session comparisons (r > 0.70 for SEMG, r > 0.77 for FW). Hence the values indicate a high reproducibility within the scope of these replication tests for the kicks. However, it needs to be emphasized that the tests only referred to one participant and, thus, are only valid to give a general orientation for the grade of reproducibility in this context.

A more detailed view to the correlation coefficients gives insight that the values of the Fd were consistently the highest throughout all the tests (r > 0.92 in the mean of intra-session comparisons and r > 0.91 in the mean of inter-session comparisons), whereas the Fp values were by far the lowest (r > 0.56 [intra-session] and r > 0.42 [inter-session]). This seems to correspond with the fact that during the *kicks* the Fd was *consistently* activated very late (short before the moment of impact), whereas the activation characteristics in Fp were more variable within the tested movements.

### Interpretation of the sequencing patterns in FW and SEMG

Concerning the comparison of *onset succession patterns* (*sequencing patterns*) within the different movement tasks over all participants, *both in FW and SEMG data*, the patterns showed that in *kicks*, the distal onsets were significantly later than in *deceleration movements and passive accelerations* (during decelerations and passive accelerations the onsets were more synchronous or even inverted, compared to the kicks).

Thus, at this point it can be stated that FW and SEMG results of the present study were widely in accordance to each other concerning *direction and context-specificity* of longitudinal succession patterns. However, for a more detailed interpretation of the *SEMG results* it needs to be considered to what extent crosstalk phenomena could have been influenced the data. In this context some of the most surprising findings were the huge onset differences of SEMG derivations and FW derivations over the *same cross-section areas* (proximal as well as distal; Fig 8; compare also S3, S4 and S5 Videos). This time delay was largest during the *kicks*, it was less during the *decelerations*, and it even turned to the contrary during the *passively induced movements*. Technical reasons for this delay between SEMG and FW can be widely excluded by the fact that the delay was too long and it was not a systematic rather than a task-specific effect. The fact of the *consistent* context-specific delay over *all participants* makes it *unlikely* that these SEMG/FW differences are caused by different onsets of *specific fibers within the RF*. We therefore assume that the origin of that phenomenon is generated by crosstalk signals (e.g. by the vasti muscles of the quadriceps). The assumption of crosstalk phenomena by the vasti in SEMG derivations over the RF is also addressed in other publications [31–34]. We therefore conclude that during conditions of highly dynamic movements, *SEMG alone* might not be a sufficient method to draw sure conclusions concerning the estimation of pure *RF activation*, because it seems also to include onset signals of adjacent muscle parts.

Even though also the *pure FW onsets* did mainly follow the patterns as hypothesized, some of the patterns were deviant. This could be interpreted as to be supported by the fact that the participants were not specifically used to the movements (such as high performance athletes) and therefore their coordination patterns might not have been *perfectly optimized* to the specific requirements. Further, the lack of standardization of the movements might be also a reason for higher data variability. However, as explained in the methods this approach was consciously chosen, because prior results [1] did show that the neuromuscular onset succession patterns do *not correlate* with kinematics rather than with the *intentional* context of movements (see also definition of the model). Therefore our chosen approach, *not* to strictly standardize the movements seems more appropriate to our hypothesis. It might be further considered if synchronous measures of *force data* (e.g. the impact of the kicks) could have been enabled further conclusions concerning existing relationships between the kinds of sequencing patterns and the resulting forces of impact. However, resulting forces during complex movements are determined by so many different factors, such as movement experience, appropriate timing of impact, intersegmental and intermuscular coordination etc., that force analyses might not be a valid method to allow conclusions concerning the effectiveness of intramuscular sequencing patterns in this context (all the more in *unexperienced* participants). Consequently, a resulting limitation for the interpretation of the present results is the fact that the results cannot provide *direct evidence* that the identified sequencing patterns are indeed more *effective* than any synchronous or even inverted activation patterns between proximal and distal muscle parts. However, because the identified patterns were induced by the intention of *maximum effectiveness*, and also our prior studies with high level athletes resulted in the same context-specific intermuscular patterns during more than 20 different kinds of movement tasks around different axes ([1–4]; compare also S1 and S2 Videos) might at least *suggest* that these context-specific sequencing patterns might be an essential function to generate movements most effectively.

### Do the results support the operationalized model?

The results of our present study suggest 1) the ability of *independent* activations in proximal and/or distal muscle parts of the RF; and 2) the functional use of this ability for a context-specific intramuscular *sequencin*g between more proximal and more distal muscle parts. Both aspects are essential prerequisites for the hypothesized functions of the “inter-fiber to tendon interaction model”.

In recent publications using SEMG arrays over the RF during cyclic movements (e.g. gait, pedalling) certain patterns of central locus activation shifts have already been described [35, 36, 37, 38]. However, the functional principles behind such findings have not yet been clarified. The operationalized “inter-fiber to tendon interaction model” could in fact be able to explain and integrate these findings within a consistent functional context and could be additionally interpreted as to be in line with some of the most recent publications of Watanabe et al. during *gait* [35, 36]. Even though the authors did not describe the idea of intramuscular IMS functions (as defined by the functional principles of the Pfix-Pmob model and operationalized by the “inter-fiber to tendon interaction model”), their results of central locus activation shifts in *SEMG arrays* over the RF during gait can be also interpreted as a progressive activation pattern from prox to dist with the beginning of the *swing phase* (for co-directed positive acceleration with the foot = Pmob) and, inversely, from dist to prox with the beginning of the *stance phase* (for co-directed negative acceleration with the foot = Pfix). The side aspect of our observations during steps (compare “supplementary findings” in the results) provide interesting parallels with these findings and also seem to functionally fit with the findings during the “kicks” (“swing phase”) and “decelerations” (transition to the “stance phase”) with lower intensities.

Furthermore, according to the overlapping characteristics of *intersegmental* agonist/antagonist interaction as demonstrated in Fig 1, also the findings concerning the agonist/antagonist interaction in the *present* study seem to be in line with the operationalized model. The specific overlapping of agonist’s *proximal* fibers *against* the antagonist’s resistance could functionally enhance (and/or control) the extent and timing of straightening the aponeurosis and tendon and thereby support the “*longitudinal catapult effect*” for an effective generation of whip-like movements such as kicks. Thus, the proposed “inter-fiber to tendon interaction model” could indeed be able to *functionally* explain the phenomenon of longitudinal regional compartmentalization and to integrate the findings of the present study with results of prior publications.

Nevertheless, the results need to be interpreted carefully. Even though the findings form a first *indication* to support the mechanisms as developed in that model, pure EMG data alone are *not able to finally verify* the complex kinematics of that model. EMG does not give any information about whether the neuromuscular activation takes place during eccentric, isometric, or concentric work of the fibers, or about when and to what extent the associated elastic structures are straightened or not. Therefore, to definitely verify the model, additional measurement techniques, such as invasive measurements of the kinematics of different fibers, the aponeuroses and tendons during actively performed natural movements would be necessary. Such interventions, however, are difficult to apply in humans up to now.

Revisiting the starting point of this article and the development of our approach (compare Fig 1, S1 and S2 Videos), the findings of this study seem to *obtrude* the idea of such consistent completion of inter- and intramuscular cross-interlocking between contractile and elastic structures to generate movements most effectively. Such kind of multisystem-interlocking coordinative improvement could be an essential element of context-specific functional adaptation, to substantially improve human movement’s efficiency and to minimize metabolic cost of pure contractile work. Future investigation is necessary to further support this idea.

## Supporting information

S1 Table Intra-and inter-task analyses (linear mixed models 1 and 2). (PDF)

S2 Table Dataset. (XLS)

S3 Table Dataset. (XLS)

S1 Video Inter- and intrasegmental longitudinal sequencing (example 1). Representative example of SEMG patterns of a junior gymnast during giant swings on high bar (multisegmental co-directed rotational movements). Electrodes were placed over pectoralis mayor; upper rectus abdominis; lower rectus abdominis; upper rectus femoris; lower rectus femoris. The onset succession runs each from Pfix to Pmob including the *intra*segmental derivations over rectus abdominis and rectus femoris. Video is depicted in custom made software “Vismo”. Signals smoothed over 50ms; slow motion with half pace.
(AVI)

S2 Video Inter- and intrasegmental longitudinal sequencing (example 2). Representative example of another junior gymnast during variations of giant swings on high bar (including a dismount) with the same setup as in S1 Video. In the beginning of the video the typical onset succession from Pfix to Pmob during “normal” giant swings can be observed. In contrast to the patterns of the preceding “normal” giant swings, the latency between proximal and distal RF activation is clearly increased during the *boosted kick* for efficient leg acceleration before dismount (at 11.07 s). (Note: In contrast to most top performance athletes (compare [1, 4]) the rectus abdominis activation of this gymnast comes too late at that moment (at 11.07 s); this can result in increased pelvis tilt to hyperlordosis).
(AVI)

S3 Video Kick. Representative example of the EMG patterns during a kick. Signals smoothed over 50ms (RMS); Pace in slow motion with a tenth of normal velocity. A very early activation of proximal FW can be observed.
(AVI)

S4 Video Deceleration after sprint. Representative example of the EMG patterns during deceleration. Signals smoothed over 50ms (RMS); Pace in slow motion with a tenth of normal velocity.
(AVI)

S5 Video Passively induced movements. Representative example of EMG patterns during passive acceleration. Signals smoothed over 50ms (RMS); normal pace.
(AVI)

S6 Video Examples of isolated activations. Example of EMG patterns during the preparation between measurements. Video in slow motion with half pace of normal velocity.
(AVI)

S7 Video Sequencing patterns during a step. The example shows a “proximal to distal activation” during the swing phase and a “distal to proximal activation” in the beginning of the stance phase. Video in slow motion with a tenth of normal velocity.
(AVI)
