# Supplementary material for: Longitudinal sequencing in intramuscular coordination: A new hypothesis of dynamic functions in the human rectus femoris muscle
Source: PLoS One. 2017 Aug 17;12(8):e0183204. doi: 10.1371/journal.pone.0183204 (PMC5560678; doi:10.1371/journal.pone.0183204)
Supplement: S1 Table — (PDF) [file pone.0183204.s001.pdf]

S1 Table: Intra-task and inter-task analyses

Table A: Intra-task analysis (linear mixed model 1)

	Descriptive data				Inferential analysis				
	Mean	SD	Cohen´s d	Succession (in %)	SE	DF	t-value	p-value	95%-CI (lower / upper)
**Kicks**									
Fp-Fd	0.065	0.124	0.52	69.2 (Fp-Fd)	0.029	12	2.22	**0.046**	0.00 / 0.13
Sp-Sd	0.134	0.075	1.79	100 (Sp-Sd)	0.016	12	8.13	**<0.001**	0.10 / 0.17
Sp-Sm	0.132	0.076	1.74	100 (Sp-Sm)	0.017	12	7.86	**<0.001**	0.10 / 0.17
Sm-Sd	0.002	0.008	0.25	58.9 (Sm-Sd)	0.001	12	0.23	0.227	0.00 / 0.00
Sp-Fp	0.199	0.097	2.05	97.4 (Sp-Fp)	0.020	12	10.00	**<0.001**	0.16 /0.24
Sd-Fd	0.130	0.070	1.86	100 (Sd-Fd)	0.018	12	7.44	**<0.001**	0.09 /0.17
**Decel.**									
Fp-Fd	-0.038	0.037	1.03	88.2 (Fd-Fp)	0.010	11	-3.63	**0.004**	-0.06 /-0.01
Sp-Sd	-0.009	0.035	0.26	58.8 (Sd-Sp)	0.010	11	-0.49	0.632	-0.03 / 0.02
Sp-Sm	0.003	0.032	0.09	64.7 (Sp-Sm)	0.009	10	0.68	0.510	-0.01 / 0.03
Sm-Sd	-0.011	0.016	0.69	72.5 (Sd-Sm)	0.004	11	-2.71	**0.020**	-0.02 / 0.00
Sp-Fp	0.033	0.030	1.1	88.2 (Sp-Fp)	0.009	10	3.84	**0.003**	0.01 /0.05
Sd-Fd	0.004	0.047	0.09	64.7 (Sd-Fd)	0.013	12	0.18	0.859	-0.03 /0.03
**Passive**									
Fp-Fd	-0.016	0.042	0.38	65 (Fd-Fp)	0.009	13	-1.58	0.137	-0.03 / 0.01
Sp-Sd	-0.004	0.018	0.02	50 (Sd-Sp)	0.003	13	-1.36	0.197	-0.01 / 0.00
Sp-Sm	0.000	0.014	0	52.5 (Sm-Sp)	0.003	13	-0.01	0.991	-0.01 / 0.01
Sm-Sd	-0.004	0.024	0.17	60 (Sd-Sm)	0.005	13	-0.91	0.378	-0.01 / 0.01
Sp-Fp	-0.015	0.040	0.38	57.5 (Fp-Sp)	0.007	12	-2.16	**0.050**	-0.03 / 0.00
Sd-Fd	-0.026	0.055	0.47	67.5 (Fd-Sd)	0.011	13	-2.34	**0.036**	-0.05 / 0.00

Fp: fine wire proximal derivation; Fd: fine wire distal derivation; Sp: SEMG proximal derivation; Sm: SEMG middle derivation; Sd: SEMG distal derivation; Decel: deceleration movements; Passive: passive movements; SD: standard deviation; SE: standard error; DF: degrees of freedom; CI: confidence interval. Significant p-values are printed fat (p-values represent original values and are **not** corrected by alpha-correction procedures).

Table B: Inter-task analysis (linear mixed model 2)

	Grouping (I)	Grouping (J)	Means (I-J)	SE	DF	p-value	95% CI (lower / upper)
**Fp-Fd**	Kicks	Decel.	0.102	0.030	45.102	**0.004**	0.028 / 0.176
		Passive	0.079	0.026	34.542	**0.015**	0.013 / 0.146
	Decel.	Passive	0.023	0.029	45.474	1.000	-0.050 / 0.095
**Sp-Sd**	Kicks	Decel.	0.140	0.017	46,117	**<0.001**	0.096 / 0.183
		Passive	0.138	0.015	32.890	**<0.001**	0.099 / 0.177
	Decel.	Passive	-0.002	0.017	47.067	1.000	-0.045 / 0.041
**Sp-Sm**	Kicks	Decel.	0.127	0.017	46.139	**<0.001**	0.084 / 0.170
		Passive	0.132	0.015	33.341	**<0.001**	0.094 / 0.171
	Decel.	Passive	0.005	0.017	47.078	1.000	-0.038 / 0.048
**Sm-Sd**	Kicks	Decel.	0.013	0.006	47.778	0.086	-0.001 / 0.027
		Passive	0.006	0.005	31.139	0.716	-0.006 / 0.018
	Decel.	Passive	-0.007	0.006	48.787	0.657	-0.021 / 0.007
**Fp-Sp**	Kicks	Decel.	-0.165	0.022	50.148	**<0.001**	-0.220 / -0.111
		Passive	-0.214	0.019	33.016	**<0.001**	-0.262 / -0.167
	Passive	Decel.	0.049	0.022	51.146	0.093	-0.006 / 0.103
**Fd-Sd**	Kicks	Decel.	-0.128	0.022	42.444	**<0.001**	-0.182 / -0.074
		Passive	-0.158	0.019	33.901	**<0.001**	-0.207 / -0.109
	Passive	Decel.	0.030	0.021	43.229	0.510	-0.023 / 0.083

Fp: fine wire proximal derivation; Fd: fine wire distal derivation; Sp: SEMG proximal derivation; Sm: SEMG middle derivation; Sd: SEMG distal derivation; Decel: deceleration movements; Passive: passive movements; SE: standard error; DF: degrees of freedom; CI: confidence interval. Significant p-values are printed fat (post-hoc pairwise comparison with Bonferroni correction was applied).
